# Hospital admissions and deaths relating to deliberate self-harm and accidents within 5 years of a cancer diagnosis: a national study in Scotland, UK

**DOI:** 10.1038/sj.bjc.6603617

**Published:** 2007-02-13

**Authors:** D R Camidge, D L Stockton, S Frame, R Wood, M Bain, D N Bateman

**Affiliations:** 1 University of Edinburgh, Edinburgh Cancer Centre, Western General Hospital, Crewe Road, Edinburgh EH4 2XU, UK; 2 Information Services, NHS National Services Scotland, Gyle Square, 1 South Gyle Crescent, Edinburgh EH12 9EB, UK; 3 Scottish Poisons Information Bureau, Royal Infirmary of Edinburgh, Little France Crescent, Edinburgh EH16 4SA, UK

**Keywords:** suicide, deliberate self-harm, accident, relative risk

## Abstract

The risk of suicide in cancer patients has been reported as elevated in several countries. These patients are exposed to many medicines that may confuse or provide a means for harm, potentially also increasing their risk from accidents. Ratios of observed/expected numbers of hospital admission and death events relating to deliberate self-harm (DSH) and accidents were calculated in the 5 years from a cancer diagnosis in Scotland 1981–1995, compared to the matched general population. The relative risk (RR) of suicide was 1.51 (95% confidence interval (CI): 1.29–1.76). The RR of hospital admissions for DSH was not significantly increased, suggesting a strong suicidal intent in DSH acts in cancer patients. Accidental poisonings and all other accidents were both increased (RR death=3.69, 95% CI: 2.10–6.00; and 1.58, 95% CI: 1.48–1.69, respectively) (RR hospital admissions=1.32, 95% CI: 1.19–1.47; and 1.55, 95% CI: 1.53–1.57, respectively). The association of only certain tumour types (e.g. respiratory) with suicide and accidental poisoning, and a broad range of tumour types with an elevated risk of all other accidents, suggests accidental poisoning categories may be a common destination for code shifting of some DSH events. A previous history of DSH or accidents, significantly increased the RR of suicide or fatal accidents, respectively (RR suicide=14.86 (95% CI: 4.69–34.97) *vs* 1.16 (95% CI: 0.84–1.55)) (RR accidental death=3.37 (95% CI: 2.53–4.41) *vs* 1.29 (95% CI: 1.12–1.49)). Within 5 years of a cancer diagnosis, Scottish patients are at increased RR of suicide and fatal accidents, and increased RR of hospital admissions for accidents. Some of these accidents, particularly accidental poisonings, may contain hidden deliberate acts. Previous DSH or accidents are potential markers for those most at risk, in whom to target interventional techniques.

Patients diagnosed with cancer are often on multiple different medications, many of them with the potential to confuse and/or provide a means for harm. In conjunction with other causes of confusion or debility relating to their underlying disease, their risk of accidental harm may be increased. Similarly, depression and suicidal or deliberate self-harm (DSH) ideation are known to be common within this patient group (Chochinov, 2001), which may increase their risk of non-fatal DSH and of fatal DSH (suicide) (Louhivuori and Hakama, 1979; Fox *et al*, 1982; Allebeck *et al*, 1989; Allebeck and Bolund, 1991; Levi *et al*, 1991; Crocetti *et al*, 1998; Tanaka *et al*, 1999; Innos *et al*, 2003; Yousaf *et al*, 2005).

All events resulting in death or hospital admission within the UK are encoded within databases according to the version of the International Classification of Diseases (ICD) relevant at the time (Camidge *et al*, 2003). When it is impossible to determine whether the motivation behind an act of harm was accidental or deliberate, undetermined intent event categories may be used instead.

Low socioeconomic status has been associated with both suicidal behaviour and a higher incidence of several common tumour types, but has never been addressed as a potential confounder of any change in relative risk (RR) of DSH in cancer patients (McLoone and Boddy, 1994; Gunnell *et al*, 1995). Although an increased risk of suicide among those diagnosed with cancer (either when codified as DSH, or when DSH events are assessed in combination with events of undetermined intent) has already been noted in a number of countries and/or regions of countries, no studies on the RR of suicide or fatal accidental harm in UK cancer patients have previously been reported. In addition, there has never previously been a national study from any country on hospital admissions for DSH or on accidents in cancer patients. Although past DSH is a well-recognised risk factor for completed suicide in the general population, the impact of this, and of past accidental harm, on the RR of harm following a cancer diagnosis has also never been reported previously. Scotland is exceptional internationally, and unique among the constituent countries of the UK because individual patient identification is possible within the high-quality linked national databases allowing RRs of death and hospital admissions, socioeconomic status (deprivation category) adjustments, repeat admissions and hospital admissions preceding deaths, all to be assessed on a countrywide scale.

The RRs of deaths and hospital admissions for DSH events, and of accidents, among cancer patients were quantified compared to the general Scottish population adjusted by age, sex, calendar year of diagnosis and deprivation category. The effect of time from the cancer diagnosis on the risk of harm, and on the methodologies used for the harm, was also assessed. Specific risk factors including previous history of harm, gender, tumour type and year of diagnosis were explored in subgroup analyses.

## METHODS

Information Services of NHS Scotland (ISD) produces a data set containing SMR1/01 (Scottish Morbidity Records 1), SMR6 (Cancer Registration Records) and Registrar General's death records, on over 5.8 million patients, linked together using ‘probability matching.’ It is estimated that the probability-matching algorithm, which uses all available identifying information (e.g. name, date of birth, postcode, hospital reference number), links these records with an accuracy of 98% (Kendrick and Clarke, 1993). The SMR1/01 database covers all non-obstetric and non-psychiatric discharges from NHS hospitals in Scotland.

From the linked data set we identified all patients, aged 15 years or more, diagnosed with invasive cancer, excluding non-melanomatous skin cancer, between 1981 and 1995. Patients diagnosed from death certificate only were excluded from the analysis. If a patient was diagnosed with >1 cancer, only the first was used as an index lesion. Subsequent SMR1 entries or deaths codified as DSH, event of undetermined intent or an accident were identified within 5 years of the cancer diagnosis (see Table 1 for detailed ICD codes used). Owing to apparent inconsistencies in the coding of accidental events within the secondary death field over time, only the primary cause of death was considered. For the hospital admissions data, both primary and secondary field information were used. Accidents were subdivided for analysis into two separate bespoke categories: accidental poisonings and all other accidents excluding accidental poisonings.

To determine the expected number of events, the person-years at risk for the cancer cohort(s) summed within each calendar year, by age (5 year age bands) and by sex were calculated from the date of each cancer diagnosis to the date of death (or hospital admission) or to 5 years from the incidence date, whichever occurred first. Additional person-year adjustments by deprivation category were explored, using postcode-derived Carstairs quintiles from the 1991 census (Morris and Carstairs, 1991). The year-, age-, sex- (and deprivation category-) specific rates of in-patient admissions and deaths codified as DSH, undetermined intent or accidents in Scotland were then applied to the cancer cohort person-years at risk. Relative risk was defined as the ratio of observed/expected. Events codified as either DSH or of undetermined intent were combined in the analyses, unless stated otherwise. Initial analyses revealed an anomalously large excess RR of death by accidental poisoning among SMR6 mesothelioma cases. This appeared to be because of a specific problem with coding deaths from mesothelioma in the Registrar General death records (Camidge *et al*, 2006); therefore before the final analyses, mesotheliomas were removed from both the cancer cohort and the general population controls. Ninety-five percent confidence intervals (CIs) for RR were estimated assuming the observed numbers followed a Poisson distribution.

Selected subgroup analyses of specific risk factors based on tumour type, age, deprivation index, gender and calendar year of diagnosis were explored. To assess the impact of previous harm-related behaviour on the RR of deaths from suicide and accidents after a diagnosis of cancer, RRs were explored in those patients diagnosed, 1991–1995, with and without a history of hospital admissions for harm-related behaviour in the 10 years before the cancer diagnosis.

## RESULTS

### Characteristics of confirmed suicides

The population of Scotland is approximately 5.1 million. Between 1981 and 1995, 315 041 Scottish residents aged 15 years or more were diagnosed with invasive cancer (excluding non-melanomatous skin cancer and mesothelioma). Of these, 131 died and were codified as suicides (DSH) (82 males, 49 females) within 5 years of their index cancer registration date (i.e. 1981–2000). A total of 11 450 members of the general Scottish population (aged 15 years or more) were codified as committing suicide (DSH) over the same time period (1981–2000).

The suicide methods used in the unmatched general Scottish population, 1981–2000, and by those in the cancer cohort, 1981–2000, are shown in Table 2. Changes with time from the cancer diagnosis are shown in Table 3. Pooled data regardless of the precise suicide method used were employed for all subsequent analyses.

### Relative risk of death codified as DSH and/or undetermined intent, or accidents

After adjusting for age, sex, calendar year and deprivation category there was an excess RR of death by suicide (DSH and events of undetermined intent combined) (RR=1.51, 95% CI: 1.29–1.76), by accidental poisoning (RR=3.69, 95% CI: 2.10–6.00) and by all other accidents combined (RR=1.58, 95% CI: 1.48–1.69) in the cancer cohort compared to the matched general population in Scotland (Table 4). Repeating the analyses without matching by deprivation category made little difference to the results (Table 4). Full matching was used throughout the exploration of specific risk factors (Table 6).

### Relative risk of hospital admission codified as DSH and/or undetermined intent, or accidents

After adjusting for age, sex, calendar year and deprivation category there was an excess RR of hospital admissions for accidental poisoning (RR=1.32, 95% CI: 1.19–1.47) and for all other accidents, excluding accidental poisoning (RR=1.55, 95% CI: 1.53–1.57) in the cancer cohort compared to the matched general Scottish population (Table 5). There was no excess risk of hospital admissions codified as DSH or events of undetermined intent. Again, analyses not matched by deprivation category had very similar results. Full matching was used throughout the exploration of specific risk factors (Table 6).

The RR of death subdivided by presence/absence of hospital admissions for harm within 10 years preceding the cancer diagnosis is shown in Table 7.

## DISCUSSION

In Scotland, the RR of suicide (DSH and undetermined intent events combined) within 5 years of a diagnosis of cancer was 1.51 (95% CI: 1.29–1.76) times that of the fully matched general population (Table 4). For males, the RR was 1.38 (95% CI: 1.12–1.68), and for females it was 1.74 (95% CI: 1.36–2.19) (Table 6). Previous studies conducted in non-UK countries, or regions of countries, have demonstrated similarly altered RRs of suicide (either when codified as DSH, or when DSH events are assessed in combination with events of undetermined intent) for male cancer patients in the 1.3–2.8 range, and for female cancer patients in the 0.5–2.7 range (Louhivuori and Hakama, 1979; Fox *et al*, 1982; Allebeck *et al*, 1989; Allebeck and Bolund, 1991; Levi *et al*, 1991; Crocetti *et al*, 1998; Tanaka *et al*, 1999; Innos *et al*, 2003; Yousaf *et al*, 2005).

All such studies, based on ratios of observed/expected events, are likely to contain certain systematic errors. For example, the inclusion of data from cancer patients as a small proportion of the data within the general population rates used to generate the expected numbers of events. Within our own study, unquantifiable errors or omissions, that are also likely to be small, will have included missing any non-fatal accidents or DSH that do not present to hospital, and similarly missing patients who leave the area of record, in this case Scotland, before experiencing harm-related events.

In addition, it is widely accepted that acts of DSH may not be coded as such in hospital admission or death records, either through genuine uncertainty about the motivation behind the act, or perhaps through a desire to spare the patient or family any perceived stigma associated with a label of DSH (Linsley *et al*, 2001; Rhodes *et al*, 2002). The extent to which such ‘code shifting’ occurs, or differs between countries, is unknown. We deliberately included events of undetermined intent in with DSH events to partially address this. In Scotland, when analysed separately the inclusion/exclusion of undetermined intent deaths in with DSH made only a small difference to the RR of death from suicide, (RR of suicide without inclusion of undetermined intent events=1.61 (95% CI: 1.28–2.00) in males and 1.68 (95% CI: 1.24–2.22) in females).

There was a trend towards the numbers of suicides dropping over time from the cancer diagnosis (Table 3). Similar patterns have been reported in many of the non-UK cancer-suicide studies consistent with the greatest psychological morbidity occurring on diagnosis (Louhivuori and Hakama, 1979; Fox *et al*, 1982; Allebeck *et al*, 1989; Allebeck and Bolund, 1991; Levi *et al*, 1991; Storm *et al*, 1991; Crocetti *et al*, 1998; Tanaka *et al*, 1999; Innos *et al*, 2003; Yousaf *et al*, 2005). Suicide in the setting of cancer may, for example, be more associated with hopelessness than depression *per se*. We had hypothesised that increasing access to pharmaceuticals may affect the methods used for suicide among cancer patients, but found only minor differences in the overall methods used compared to the general population (Table 2). However, the proportions in each time period (<1, 1–2 and 3–5 years from diagnosis) choosing poisoning (solids, liquids or gases) *vs* more violent/other means did suggest a trend towards more pharmaceutically driven methodologies over time, perhaps owing to increased use of some of these medications associated with disease progression (37 *vs* 63, 52 *vs* 48 and 50 *vs* 50%, respectively) (Table 3). Fatal accidents and accidental poisonings were also increased among cancer patients compared to the matched general population (Table 4).

The records used for hospital admissions do not contain purely non-fatal data. However, as the percentage of cancer cohort hospital admissions resulting in fatal events during the same stay, and codified under the same category, was ⩽3%, they appeared a reasonably surrogate for non-fatal harm within our data sets. The RR of hospital admission was increased for both accidental poisonings and all other accidents, excluding accidental poisoning, but was not significantly altered for hospital admissions for DSH or events of undetermined intent (Table 5). Although some code shifting may have occurred, as the primary witness to the act in non-fatal events is still alive, the intention behind the act in hospital admissions is perhaps more likely to be accurately ascribed than with the death data. The fact that the RR of hospital admissions for DSH and/or events of undetermined intent was not altered among cancer patients, in contrast to their elevated RR of suicide, suggests that acts of DSH in cancer patients may be associated with stronger suicidal intent than in the general population. However, proportions of completed suicide are only a very crude way of assessing suicidal intent as patients with high suicidal intent may not succeed in their attempts and vice versa. In the only previous study looking at suicide attempts, the RR for a non-fatal hospital admission with DSH or an event of undetermined intent in Stockholm County, Sweden was elevated for both male (1.7, 95% CI: 1.4–2.1) and female cancer patients (1.3, 95% CI: 1.1–1.6). However, the overall pattern of these RRs being substantially lower than for fatal acts in the same population was similar to that seen in the Scottish national data (Allebeck and Bolund, 1991).

We found no clear discernible pattern of altered RR by age group or deprivation index (data not shown), or by calendar year of cancer diagnosis (5 years bands) (Table 6). Respiratory, digestive tract and female genital tract tumours were particularly associated with a statistically significantly increased RR of suicide, and respiratory and head and neck tumours with hospital admissions for DSH/events of undetermined intent (Table 6). Apparent tumour-type-specific risks are inevitably confounded by a number of variables not matched for but potentially associated with predispositions to accidental or DSH, such as alcohol or tobacco abuse, because of the influence of these factors on the incidence of the different tumour types. In addition, RRs are more likely to reach statistical significance for common than for rare tumours/presentations, purely because of the larger number of individuals involved. Nevertheless, the association of specific tumour types, particularly respiratory and head and neck tumours, and not other common tumours, for example, breast, with not only DSH-related events but deaths and/or hospital admissions relating to accidental poisoning is intriguing. Either these tumour types are genuinely more prone to accidental poisoning, or, in line with their increased DSH-related risks, that these events may not be truly ‘accidental’ and in fact represent examples of code shifting of deliberate events in high-risk groups. In contrast, the elevated risk of death or hospital admission from all other accidents across a large number of tumour types supports these categories representing mostly genuine accidents (Table 6).

No previous studies have controlled for the effect of social deprivation as a potential confounder (deprivation being associated with both self-harm and cancer) (McLoone and Boddy, 1994; Gunnell *et al*, 1995), but the presence/absence of additional matching by Carstairs deprivation index had little effect on the RRs generated from the Scottish data set (Tables 4 and 5).

In terms of absolute numbers affected, among cancer patients diagnosed in Scotland between 1981 and 1995, during the observation period 1981–2000, there were 58 more suicides and deaths of undetermined intent, and 315 more accidental deaths (all types) than expected (Table 4). Among those diagnosed with cancer, during the same observation period (1981–2000) there were 7666 more hospital admissions for accidental harm (all types) than expected (Table 5).

Looking only at those patients diagnosed with cancer between 1991 and 1995, when these patients were categorised by whether or not they had a previous history of non-fatal DSH/event of undetermined intent within the 10 years before their cancer diagnosis, the RRs of suicide appeared strikingly different at 14.86 (95% CI: 4.69–34.97) and 1.16 (95% CI: 0.84–1.55), respectively (Table 7). To accommodate the time period before the cancer diagnosis, the numbers involved in these analyses were smaller than within the main analysis and as a consequence the CIs around each RR are larger. The RR of death by either accidental poisoning or from all other accidents was significantly elevated in those without a preceding history of harm-related behaviour, suggesting that the diagnosis of cancer *per se* is indeed associated with an additional increase in accident proneness. However, a previous history of accidental behaviour was significantly associated with a higher RR of death from all accidents (excluding accidental poisoning), suggesting that those who are accident-prone before the diagnosis of cancer, become even more so afterwards. There was also a non-statistically significant trend towards a very much greater RR of death from accidental poisoning, but not other accidents, among those with a previous history of DSH (RR=105.29; 95% CI: 0.04–604), than in those without a history of such harm-related behaviour (Table 7). In line with our other data, this again suggests that accidental poisoning may be a more likely destination than other accident categories for code shifting from truly deliberate events.

Of note, because there was no temporally defined event, comparable to the cancer diagnosis, within the general population to explore non-fatal harm events in the 10 years ‘previous to’, these risks are not compared to the general Scottish population matched by presence/absence of a previous history of harm. Consequently, these data cannot determine whether the diagnosis of cancer and a history of harm are independent risk factors. To resolve this, more exhaustive studies, for example, involving cohorts grouped at a given time point by doublets of cancer/harm, no cancer/harm, cancer/no harm and no cancer/no harm with prospective follow-up would be required. Whatever the precise explanations, the identification of previous accidents or DSH as a marker for those most at risk of DSH or accidental harm following a diagnosis of cancer is the strongest indication to date of a particular group of cancer patients in which to target interventional techniques. Outpatient-based depression and anxiety screening and intervention has recently been piloted in the oncology setting in a number of hospitals within the UK (Sharpe *et al*, 2004). Based on these data, we would suggest that asking about, or identifying through medical records, a previous history of DSH or accidental harm should be explored as part of any such screen of oncology patients in the future. Although psychological and psychiatric intervention may be appropriate for those identified with an elevated risk of DSH – or of events likely to represent hidden acts of DSH – focussed home visits by key professionals, such as occupational therapists, general practitioners and district nurses, may also be required to affect the elevated risk of genuine accidents in this vulnerable population.

## Figures and Tables

**Table 1:** ICD codes of harm events used in analysis

**Description**	**ICD9 code^a^**	**ICD10 code^b^**
Deliberate self-harm	E950–E959	X60–X84, Y87.0
Event of undetermined intent	E980–E989	Y10–Y34, Y87.2
Accident group A (all accidents – excluding accidental poisoning)	E800–E849, E870–E949	V01–V99, W00–W99, X00–X39, X50–X59, Y40–Y59, Y85, Y86
Accident group B (accidental poisoning)	E850–E869	X40–X49

a Up to 31 December 1999 for deaths and up to 31 December 1996 for hospital admissions.

b From 1 January 2000 for deaths and from 1 January 1997 for hospital admissions.

**Table 2:** Breakdown of suicides by methodology of act, within 5 years of a cancer diagnosis made between 1981 and 1995 (i.e. 1981–2000) and in the (unmatched) general Scottish population between 1981 and 2000

	**Scottish population**		**Cancer cohort**	
**Type of suicide**	***n***	**%**	***n***	**%**
*Solids or liquids*	3261	28.5	42	32.1
Analgesics, antipyretics and anti-rheumatics	1157	10.1	17	13
Tranquillizers and other psychotropics	1027	9.0	12	9.2
Other	1077	9.4	13	9.9
Gases	1604	14.0	17	13
Hanging	3644	31.8	22	16.8
Drowning	1010	8.8	19	14.5
Firearms	456	4.0	7	5.3
Cutting	167	1.5	6	4.6
Jumping	730	6.4	13	9.9
Other/unspecified	578	5.0	5	3.8
				
Total	11 450	100	131	100

**Table 3:** Methodology of suicide in cancer patient cohort by time since cancer diagnosis

		**Time to death from cancer diagnosis**			
**Type of suicide**		**<1 year**	**1–2 years**	**3–5 years**	**Total**
Analgesics, antipyrectics and anti-rheumatics	n	4	5	8	17
	%	24%	29%	47%	100%
Tranquillizers and other psychotropics	n	4	5	3	12
	%	33%	42%	25%	100%
Other solids or liquids	n	5	1	7	13
	%	38%	8%	54%	100%
Gases	n	6	3	8	17
	%	35%	18%	47%	100%
Hanging	n	12	2	8	22
	%	55%	9%	36%	100%
Drowning	n	6	5	8	19
	%	32%	26%	42%	100%
Firearms	n	4	1	2	7
	%	57%	14%	29%	100%
Cutting	n	2	2	2	6
	%	33%	33%	33%	100%
Jumping	n	8	3	2	13
	%	62%	23%	15%	100%
Other/unspecified	n	1	0	4	5
	%	20%	0%	80%	100%
Total	n	52	27	52	131
	%	40%	21%	40%	100%

Percentages rounded to nearest whole number.

**Table 4:** Relative risk of death codified as DSH, undetermined intent or accidents in cancer patients compared to the matched general Scottish population after adjusting for age, sex and calendar year +/− deprivation category

**Deaths**	**Observed**	**Expected**	**Relative risk**	**Lower 95% CI**	**Upper 95% CI**
*Adjusted for year, age, sex*					
Deliberate self-harm and undetermined intent	172	115.5	1.49	1.28	1.73
Accidental poisoning	16	4.4	3.67	2.09	5.97
All accidents excluding accidental poisoning	826	530.9	1.56	1.45	1.67
					
*Adjusted for year, age, sex, deprevation*					
Deliberate self-harm and Undetermined intent	172	113.8	1.51	1.29	1.76
Accidental poisoning	16	4.3	3.69	2.10	6.00
All accidents excluding accidental poisoning	826	522.3	1.58	1.48	1.69

**Table 5:** Relative risk of hospital admission codified as DSH, undetermined intent or accidents in cancer patients compared to the matched general Scottish population after adjusting for age, sex and calendar year +/− deprivation category

**Hospital admissions**	**Observed**	**Expected**	**Relative risk**	**Lower 95% CI**	**Upper 95% CI**
*Adjusted for year, age, sex*					
Deliberate self-harm and Undetermined intent	794	804.5	0.99	0.92	1.06
Accidental poisoning	356	276.4	1.29	1.16	1.43
All accidents excluding accidental poisoning	21 592	14 622.2	1.48	1.46	1.50
*Adjusted for year, age, sex, deprivation*					
Deliberate self-harm and undetermined intent	794	761.3	1.04	0.97	1.12
Accidental poisoning	356	268.7	1.32	1.19	1.47
All accidents excluding accidental poisoning	21 592	13 946.2	1.55	1.53	1.57

Note: Owing to the inclusion of both primary and secondary field information in the hospital admission data, a single event resulting in admission may appear within >1 subcategory of harm.

**Table 6:** Relative risks of death and hospital admission within 5 years of a cancer diagnosed, 1981–1995, compared to the matched general Scottish population subdivided by tumour type, sex and calendar year of cancer diagnosis

	**Death within 5 years of cancer diagnosis, 1981–1995 (95% CIs)**					
**Subgroups**	**DSH+undetermined**		**Accidental poisoning**		**All accidents excluding accidental poisoning**	
Overall	1.51	1.29–1.76	3.69	2.1–6	1.58	1.48–1.69
Head and neck	1.49	0.53–3.26	6.95	0–39.8	1.67	1.1–2.43
Digestive	1.58	1.12–2.19	1.14	0–6.56	1.56	1.36–1.79
Respiratory	1.71	1.08–2.57	8.03	2.09–20.8	2.44	2.01–2.94
Breast	1.37	0.87–2.07	4.17	0.79–12.3	1.28	1.07–1.53
Female genital	2.52	1.46–4.04	3.30	0–18.9	1.52	1.1–2.06
Prostate	1.33	0.77–2.13	4.59	0.43–16.9	1.12	0.88–1.41
Male genital	1.12	0.29–2.89	—	—	3.39	1.89–5.61
Urinary	1.11	0.63–1.81	3.87	0.36–14.2	1.34	1.07–1.66
Skin	1.16	0.37–2.73	—	—	0.45	0.18–0.94
Lymphoid	1.47	0.8–2.47	—	—	2.42	1.94–2.99
Secondaries/unspec	2.72	0.51–8.04	22.37	0.01–128.2	4.26	2.83–6.16
Other	1.87	0.74–3.87	6.69	0–38.4	1.61	0.92–2.62
Males	1.38	1.12–1.68	4.41	2.19–7.92	1.50	1.35–1.66
Females	1.74	1.36–2.19	2.71	0.85–6.37	1.65	1.5–1.81
Diagnosed 1981–1985	1.40	1.04–1.85	1.76	0.33–5.21	1.59	1.4–1.79
Diagnosed 1986–1990	1.73	1.33–2.22	5.04	2–10.5	1.59	1.41–1.79
Diagnosed 1991–1995	1.41	1.08–1.82	4.82	1.73–10.6	1.56	1.38–1.76
	**Hospital admission within 5 years of cancer diagnosis, 1981–1995 (95% CIs)**					
**Subgroups**	**DSH+undetermined**		**Accidental poisoning**		**All accidents excluding accidental poisoning**	
Overall	1.04	0.97–1.12	1.32	1.19–1.47	1.19	1.53–1.57
Head and neck	2.03	1.47–2.73	3.74	2.5–5.37	2.29	2.14–2.45
Digestive	1.15	0.95–1.38	1.39	1.07–1.78	1.71	1.66–1.75
Respiratory	1.59	1.28–1.96	2.08	1.53–2.76	1.78	1.71–1.86
Breast	0.93	0.8–1.08	1.05	0.82–1.34	1.26	1.22–1.31
Female genital	1.20	0.99–1.44	1.32	0.93–1.81	1.74	1.66–1.82
Prostate	1.28	0.92–1.74	1.46	0.94–2.16	1.23	1.17–1.29
Male genital	0.63	0.39–0.97	0.82	0.32–1.69	1.16	1–1.34
Urinary	1.02	0.78–1.31	1.26	0.86–1.8	1.33	1.27–1.39
Skin	0.46	0.28–0.7	0.54	0.21–1.12	1.10	1.01–1.2
Lymphoid	0.84	0.64–1.08	0.98	0.62–1.48	1.90	1.81–1.98
Secondaries/unspec	1.53	0.69–2.91	1.68	0.44–4.33	2.01	1.79–2.24
Other	0.78	0.53–1.1	1.22	0.66–2.05	2.16	2–2.33
Males	1.12	1–1.25	1.49	1.26–1.73	1.68	1.65–1.71
Females	1.00	0.91–1.09	1.22	1.05–1.4	1.46	1.43–1.49
Diagnosed 1981–1985	1.24	1.09–1.41	1.41	1.14–1.72	1.56	1.52–1.61
Diagnosed 1986–1990	1.10	0.97–1.25	1.21	0.98–1.47	1.51	1.47–1.54
Diagnosed 1991–1995	0.88	0.79–0.99	1.36	1.16–1.58	1.57	1.54–1.6

**Table 7:** Relative risk of death from harm-related conditions within 5 years of a cancer diagnosed, 1991–1995, compared to the matched general Scottish population, subdivided by history of non-fatal harm within the 10 years preceding the cancer diagnosis

	**Death within 5 years of cancer diagnosis, 1991–1995 (95% CIs)**		
**History of harm in the 10 years before cancer diagnosis**	**DSH+undetermined**	**Accidental poisoning**	**All accidents excluding accidental poisoning**
RR overall	1.39 (1.06–1.80)	4.80 (1.73–10.52)	1.54 (1.36–1.74)
No history of harm	1.16 (0.84–1.55)	4.48 (1.41–10.55)	1.29 (1.12–1.49)
History of harm (any)	3.46 (1.93–5.72)	7.44 (0–42.65)	3.13 (2.46–3.92)
Previous DSH or undetermined	14.86 (4.69–34.97)	105.29 (0.04–604)	—
Previous accidental poisoning	1.83 (0.48–4.74)	—	3.38 (2.53–4.43)
Previous accident (excluding accidental poisoning)	1.81 (0.47–4.68)	14.06 (0.01–80.62)	3.37 (2.53–4.41)
